# Harnessing the power of bioprinting for the development of next-generation models of thrombosis

**DOI:** 10.1016/j.bioactmat.2024.08.040

**Published:** 2024-09-05

**Authors:** Yanyan Liu, Tao Huang, Nicole Alexis Yap, Khoon Lim, Lining Arnold Ju

**Affiliations:** a School of Biomedical Engineering, The University of Sydney, Darlington, NSW, 2008, Australia; b Charles Perkins Centre, The University of Sydney, Camperdown, NSW 2006, Australia; c School of Medical Sciences, The University of Sydney, Darlington, NSW 2008, Australia; d The University of Sydney Nano Institute (Sydney Nano), The University of Sydney, Camperdown, NSW, 2006, Australia; e Heart Research Institute, Camperdown, Newtown, NSW 2042, Australia

**Keywords:** Bioprinting, Thrombosis, Microfluidics, Mechanobiology, Platelets

## Abstract

Thrombosis, a leading cause of cardiovascular morbidity and mortality, involves the formation of blood clots within blood vessels. Current animal models and *in vitro* systems have limitations in recapitulating the complex human vasculature and hemodynamic conditions, limiting the research in understanding the mechanisms of thrombosis. Bioprinting has emerged as a promising approach to construct biomimetic vascular models that closely mimic the structural and mechanical properties of native blood vessels. This review discusses the key considerations for designing bioprinted vascular conduits for thrombosis studies, including the incorporation of key structural, biochemical and mechanical features, the selection of appropriate biomaterials and cell sources, and the challenges and future directions in the field. The advancements in bioprinting techniques, such as multi-material bioprinting and microfluidic integration, have enabled the development of physiologically relevant models of thrombosis. The future of bioprinted models of thrombosis lies in the integration of patient-specific data, real-time monitoring technologies, and advanced microfluidic platforms, paving the way for personalized medicine and targeted interventions. As the field of bioprinting continues to evolve, these advanced vascular models are expected to play an increasingly important role in unraveling the complexities of thrombosis and improving patient outcomes. The continued advancements in bioprinting technologies and the collaboration between researchers from various disciplines hold great promise for revolutionizing the field of thrombosis research.

## Highlights

• This review details the mechanisms of thrombosis, and current limitations in traditional thrombosis models.
• This review highlights recent advancements in bioprinting techniques for constructing physiologically relevant vascular models.
• It demonstrates the benefits of using bioprinted vascular models to study thrombosis.

## Graphical abstract

**Figure:**
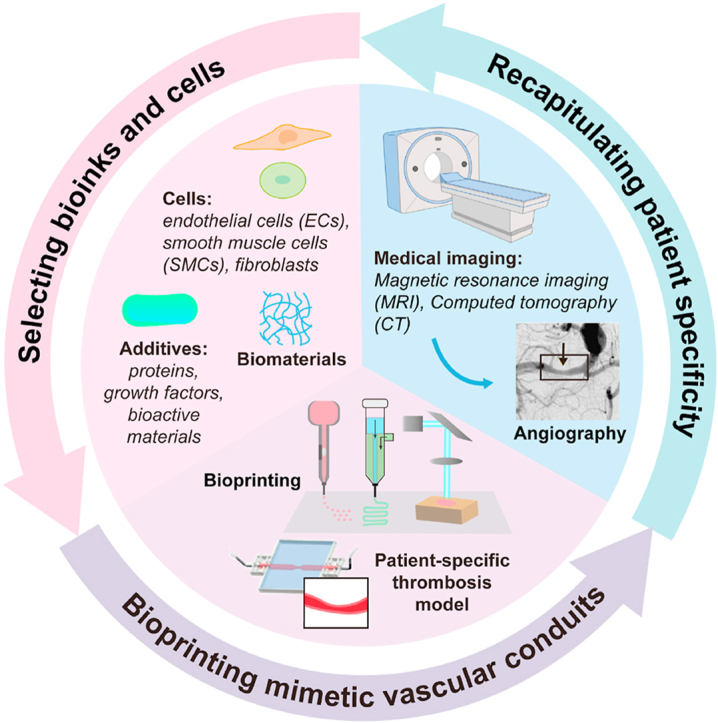

## 1 Introduction

Cardiovascular diseases (CVDs) remain the leading cause of the global disease burden. According to the Global Burden of Disease assessment and statistical analysis covering 204 countries and regions from 1990 to 2019, the total number of CVD cases nearly doubled, with the number of deaths amounting to 18.6 million people [1]. Thrombosis, the formation of blood clots within blood vessels, stands as the primary contributor to CVDs [2]. However, research on thrombosis faces challenges. The study of arterial thrombosis is constrained by the complexity and diversity of atherosclerosis, making the understanding of its mechanisms and the development of treatment methods more difficult [3]. While lifestyle and genetic factors contribute to an increased risk of venous thrombosis, its mechanisms of formation are not as well understood as those of arterial thrombosis [[4], [5], [6]]. Moreover, the conditions of thrombosis have distinct mechanisms and vary widely among individuals, depending on patient-specific factors such as vessel location, architecture, present morbidities, genetic factors, and various other characteristics. As a result, the exact mechanism of action and factors involved in pathologic thrombosis remain a major topic of research today, with many aiming to better understand the key players, risk factors, diagnostic and prognostic techniques, and new methods for combating these phenomena.

However, existing approaches for studying thrombosis involve *in vivo* animal models, which face ethical concerns and exhibit significant physiological and genetic variations compared to humans [7,8]. Although these models are costly, they are inherently limited due to differences in size, anatomy, physiology, pathology, and cardiovascular function (e.g., the average beats per minute and wall shear stresses are much greater in mice) compared to humans [9,10]. Furthermore, current *in vitro* models, particularly microfluidic models, have gained prominence as the leading state-of-the-art approach. This is attributed to their straightforward fabrication, cost-effectiveness, and ability to replicate various vascular morphologies, accommodating a broad spectrum of arterial or venous shear stresses [11]. Nevertheless, a commonly acknowledged constraint of microfluidic devices is their incapacity to faithfully replicate the intricate physiological conditions of the vascular system [12]. The materials (e.g. polydimethylsiloxane or PDMS, glass, silicon) used in microfluidic devices are different from those of blood vessel walls, potentially affecting interactions between blood components and vessel surfaces [13]. In contrast, bioprinting offers the potential to better encapsulate the shape, internal structure, and functionality of blood vessels.

**Fig. 1:**
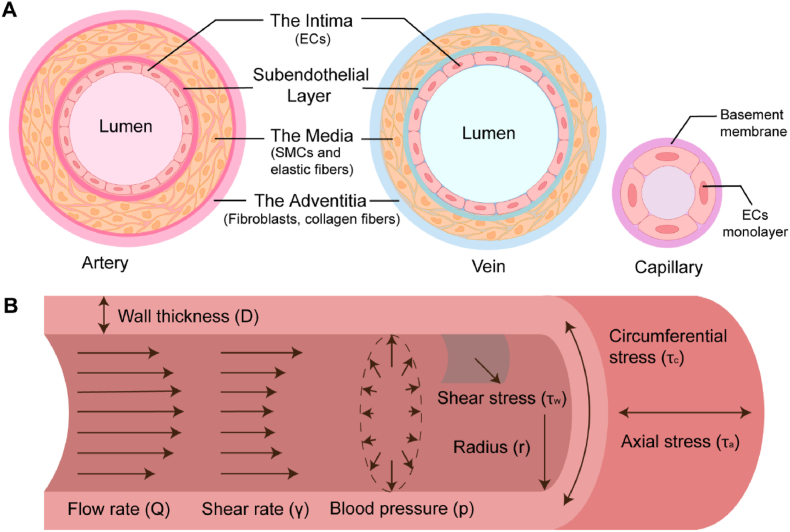
**Blood vessel wall structure and mechanical forces.** (**A**) Schematic representation of the blood vessel wall structure and composition in arteries, veins, and capillaries (not to scale). The blood vessel wall of arteries and veins consists of three layers: the intima, media, and adventitia. The intima is composed of a monolayer of ECs supported by the internal elastic lamina. The media contains SMCs and elastic fibers, while the adventitia is primarily composed of fibroblasts and collagen fibers. Capillaries consist of a monolayer of ECs and a basement membrane. (**B**) Illustration of the main mechanical forces experienced by blood vessels, including shear stress, circumferential stress, and axial stress. Shear stress (*τ*_w_) is the frictional force exerted by blood flow on the vessel wall, which is influenced by the volumetric flow rate (*Q*), shear rate (*γ*), fluid viscosity (*η*), and the radius of the lumen (*r*). Circumferential stress (*τ*_c_) is the force exerted tangentially on the vessel wall, while axial stress (*τ*_a_) is the force applied along the longitudinal axis of the vessel.

Blood vessels in their natural form comprise three layers: the intima, the media, and the adventitia, containing endothelial cells (ECs), smooth muscle cells (SMCs), and fibroblasts, respectively, all embedded within collagen and elastin (Fig. 1) [14]. The structure and components of blood vessels play a critical role in thrombosis, with alterations in vessel structure or function contributing to the development of thrombus [15,16]. Endothelial cells, in particular, are essential for maintaining vascular homeostasis and preventing thrombosis by regulating platelet activation, coagulation, and fibrinolysis [17]. Endothelial dysfunction, characterized by a shift towards a procoagulant and proinflammatory state, is a key event in the initiation of thrombosis [18]. Smooth muscle cells also contribute to the regulation of vascular tone and the maintenance of vascular integrity, with their dysfunction leading to vascular remodeling and thrombosis [19]. Understanding these relationships between vascular structure, function, and thrombosis is crucial for developing effective prevention and treatment strategies. Assessing and monitoring the process of venous thrombosis formation can help elucidate the underlying mechanisms and identify potential therapeutic targets [20]. To achieve this goal, the primary challenge of thrombosis research is to recapitulate the complex blood vessel components and structures in a physiologically relevant manner.

Bioprinting shows promise as a technology for producing customized tissue constructs, thanks to its capacity to fabricate intricate, heterogeneous structures with anatomical precision. By enabling the deposition of diverse biological components, such as growth factors, cells, genes, neo-tissues, and hydrogels resembling extracellular matrix (ECM), bioprinting offers versatile applications in tissue engineering [21]. In the investigation of venous thrombosis, bioprinting enables the construction of *in vitro* vascular conduit models that more accurately recapitulate the complex human vasculature. By incorporating key structural and mechanical characteristics of blood vessels, researchers can develop more physiologically relevant models for studying thrombosis, which offer the potential to overcome the limitations of current animal models and *in vitro* systems.

This review will discuss the challenges in thrombosis research, the importance of incorporating blood vessel complexity in bioprinted models, and the recent advancements in bioprinting techniques for constructing vascular conduits. Furthermore, the paper will highlight the application of bioprinted vascular conduits in thrombosis studies and provide insights into the future directions and challenges in this field.

## 2 Thrombosis: Mechanisms and challenges

Thrombosis, the formation of blood clots within blood vessels, is a complex process with distinct mechanisms depending on the location within the vascular system. The intricate interactions of various cellular and molecular components contribute to the pathophysiology of thrombosis. Broadly, thrombosis can be categorized into three types: arterial thrombosis, venous thrombosis, and microvascular thrombosis, each exhibiting unique characteristics and challenges in their study and treatment.

### 2.1 Arterial thrombosis

**Fig. 2:**
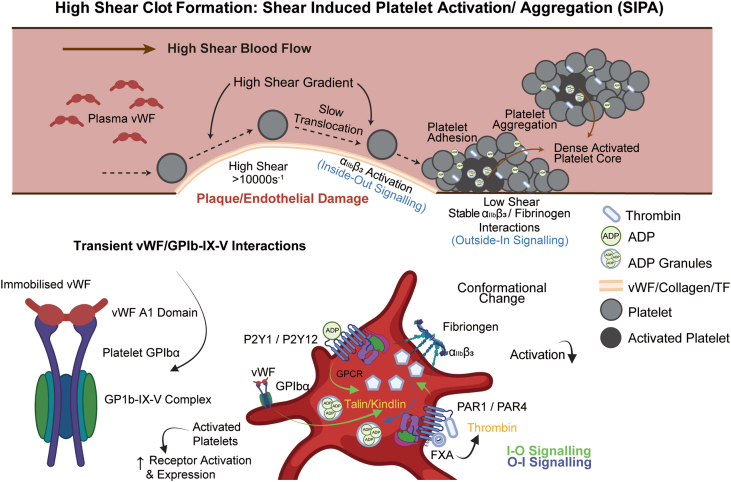
**Shear-induced platelet activation/aggregation (SIPA) and associated molecular mechanisms.** SIPA is a biological response of platelets triggered by the shear forces of blood flow, typically occurring when blood flows through stenosis or at a high shear rate (*γ*＞10,000 s^−1^) [25,26,34]. This process involves the release of vWF, changes in platelet morphology and receptor binding, platelet aggregation, and activation of intracellular signaling pathways [34]. The interaction between platelet GPIb and vWF initiates platelet adhesion and activation under high shear stress. Activated platelets release various agonists, such as ADP and TxA2, which further amplify platelet aggregation and thrombus formation. Ultimately, these events lead to the formation of a stable platelet-rich thrombus.

Arterial thrombosis occurs in the high-pressure, high-flow environment of arteries and is often associated with hypertension and atherosclerotic plaque rupture [22]. The exposed subendothelial matrix and tissue factor (TF) from the ruptured plaque initiate the coagulation cascade and platelet activation [23,24]. Platelets adhere to the exposed collagen and von Willebrand factor (vWF) through the glycoprotein (GP) Ib-IX-V complex and GPVI, leading to platelet activation and aggregation [25,26]. High shear stress in the arterial environment also contributes to platelet activation and aggregation through mechanical conduction, with shear-induced platelet activation/aggregation (SIPA) (Fig. 2) being a key mechanism driven by the interaction of platelets with immobilized vWF under high shear conditions [25,26]. Activated platelets release various agonists, such as adenosine diphosphate (ADP), thromboxane A2 (TxA2), and thrombin, which further amplify platelet aggregation and thrombus formation [27]. Additionally, platelet mechanosensing pathways involving adhesion receptors GPIb and GPIIb/IIIa play a crucial role in stabilizing formed thrombi [28,29]. Mechanobiology studies, conducted by various groups, have elucidated the various hemodynamic force effects on thrombus composition and stability [[30], [31], [32], [33]]. Bioprinted vascular models have the potential to replicate these complex mechanobiological interactions, providing insights into the pathophysiology of arterial thrombosis and potential targets for personalized therapeutic interventions.

### 2.2 Venous thrombosis

Venous thrombosis occurs in the low-pressure, low-flow environment of veins and is often associated with Virchow's triad: endothelial injury, altered hemodynamics, and hypercoagulability [35,36]. Endothelial dysfunction serves as a pivotal initial step in venous thrombosis [37]. Unlike arterial injury, which often involves plaque rupture or acute trauma, venous endothelial injury tends to be more subtle and chronic, often associated with underlying inflammatory conditions or hypoxia [38]. This dysfunction transforms the endothelium from an anticoagulant surface to a prothrombotic one, marked by the release of vWF and TF, which play crucial roles in initiating the coagulation cascade [39]. This promotes the recruitment and activation of leukocytes and platelets, which release procoagulant factors and form neutrophil extracellular traps (NETs) [40,41]. NETs provide a scaffold for thrombus formation and also contribute to the hypercoagulable state by activating factor XII and inhibiting anticoagulant pathways [42,43]. The cascade is further amplified by altered hemodynamics, a consequence of reduced blood flow velocity in veins, particularly in areas with intravascular valves or during periods of prolonged immobility. The disturbed blood flow creates an environment where platelets, leukocytes, and plasma procoagulant factors accumulate, fostering the formation of pathological clots [37]. Hypercoagulability, the third component of Virchow's Triad, refers to alterations in the composition of blood that predispose individuals to clot formation. Notably, various health factors such as age, sex, genetics, physical inactivity, obesity, surgery, infection, sepsis, intravascular implants, and fractures in the legs, hips, and pelvis can contribute to the development of venous thrombosis.

### 2.3 Microvascular thrombosis

Microvascular thrombosis occurs in the small blood vessels, such as arterioles, capillaries, and venules, and is often associated with systemic inflammation and endothelial dysfunction [44]. In conditions such as sepsis and disseminated intravascular coagulation (DIC), the widespread activation of the coagulation cascade and endothelial damage lead to the formation of microthrombi [45,46]. The microthrombi can occlude the small blood vessels, leading to tissue ischemia and organ dysfunction [44]. The pathogenesis of microvascular thrombosis involves the interplay of ECs, leukocytes, platelets, and the complement system [47,48]. NETs have been shown to play a crucial role in the development of microvascular thrombosis by promoting inflammation, platelet activation, and activation of the complement system [48]. In conclusion, microvascular thrombosis represents a complex phenomenon influenced by various factors, including vascular structure, mechanical forces, biochemical mediators, and immune responses.

### 2.4 Challenges in studying thrombosis

Studying thrombosis presents several challenges due to the complexity of the human vasculature and the diverse hemodynamic conditions within different vascular beds. The structural and functional heterogeneity of blood vessels, along with the varying shear stress profiles, can significantly influence the mechanisms of thrombosis [[49], [50], [51]]. For example, the high shear stress in the arterial system promotes platelet activation and aggregation through mechanotransduction pathways [52,53], while the low shear stress and blood stasis in the venous system favor the accumulation of procoagulant factors and the formation of fibrin-rich thrombi [37]. Additionally, the presence of valves, bifurcations, and curvatures in the vasculature can create local disturbances in blood flow, which can further contribute to thrombosis [54]. The intricate interplay between vascular architecture and hemodynamic forces underscores the need for advanced experimental models that can faithfully replicate these complexities to elucidate the underlying mechanisms of thrombosis and develop effective therapeutic strategies.

### 2.5 Limitations of current animal models and *in vitro* systems

Animal models, while valuable for understanding the *in vivo* mechanisms of thrombosis, have significant physiological and genetic differences compared to humans [7,8]. For example, the higher heart rate and shear stress in mice can lead to differences in platelet activation and thrombus formation compared to humans [9,10]. As shown in Table 1, the diameter of a mouse's artery is typically one-tenth that of a human, while the shear rate is approximately 20 times higher than that of a human [55]. These distinctions have profound pharmacogenomic implications, potentially complicating results and significantly contributing to the frequent failure of promising therapeutics as they progress to later-stage clinical trials [56].

**Table 1:** **Comparison of arterial diameter and wall shear rate between humans and mice** [55]. The table highlights the significant differences in arterial diameter and wall shear rate between humans and mice for the carotid artery, coronary artery, and aorta. The diameter of a mouse's artery is typically one-tenth that of a human, while the shear rate is approximately 20 times higher than that of a human. These distinctions have important implications for the study of thrombosis and the translation of findings from animal models to human physiology and pathology.

	Human		Murine	
	Radius (*r*, mm)	Average wall shear rate (*γ*, s^−1^)	Radius (*r*, mm)	Average wall shear rate (*γ*, s^−1^)
Carotid artery	2.15–4.1	260–500	0.15–0.25	1,800–4,000
Coronary artery	1.3–2.5	100–350	0.11	6,700
Aorta	7.0–10.0	40–150	0.35	2,500

Moreover, differences in lipidemic profiles, plaque formation, and anatomical features among animal species pose significant challenges for developing accurate CVD models and translating findings to human conditions. For example, the study of hyperlipidemia and cardioprotection lacks a suitable animal model due to differences in the lipidaemic profile of rodents compared to humans [57]. Besides, rats and dogs are particularly resistant to atherosclerotic developments [57]. Larger animals, such as dogs, pigs and non-human primates, allow for easier dissection of blood vessels, and more closely resemble the lipidaemic profile of humans [57,58]. However, the use of these animals is largely inhibited by high space and cost requirements as well as stringent ethical regulations [59]. Variations in plaque formation processes in different animal models complicate the identification of suitable animal models to study key cardiovascular disease processes. For example, lesions in rabbits consist primarily of foam cells, whereas plaque formation in pigs usually only progresses to foam cell agglomeration [59]. The different general metabolic and immunoinflammatory responses of larger animal models such as dogs also limit the clinical relevance of these models, despite their more human-like vessel diameters and treatment procedures [59]. Additionally, there also exists large inconsistencies in coronary artery systems and anatomical differences among these species. For example, rabbits lack tricuspid valves, pigs have different distributions of Purkinje fibers, and dogs possess an extensive collateral coronary system, which make observations less reliable and consistent [58,[60], [61], [62]].

Last but not least, the use of animal models also raises ethical concerns and can be costly. The FDA Modernization Act 2.0 has opened avenues for alternative approaches to strengthen the preclinical data pipeline and decrease reliance on animal models, which often lead to dead ends in therapeutic development [56]. Nevertheless, current *in vitro* surrogate systems, such as organ-on-a-chip approaches, have limitations in replicating the complex three-dimensional structure and cellular composition of native blood vessels, which can limit their ability to fully recapitulate the physiological conditions of the vascular system [11]. The materials used in microfluidic devices, such as PDMS, may not accurately represent the biomechanical properties of the vessel wall and can influence the behavior of cells and the formation of thrombi [13]. To address these challenges, there is a need for more advanced *in vitro* models that can closely mimic the structural and functional complexity of the human vasculature.

## 3 Blood vessel complexity and the need for biomimetic models

The circulatory system relies on blood vessels with diverse structures and mechanical properties to efficiently transport blood throughout the body. Vascular cells play a crucial role in maintaining a specific mechanical microenvironment [63]. However, disruptions in this delicate balance can lead to thrombosis and other cardiovascular disorders. To better understand the mechanisms of thrombosis and develop more effective therapeutic strategies, it is essential to create biomimetic models that accurately recapitulate the structural and mechanical complexity of native blood vessels. In this section, we will explore the structural diversity and mechanical characteristics of blood vessels, highlighting the importance of incorporating these features in next generation biomimetic models for thrombosis research.

### 3.1 Structural diversity of blood vessels

The blood vessel network can be broadly categorized into arteries, veins and capillaries [64]. Each with unique characteristics tailored to their respective functions. Arteries exhibit high elasticity to accommodate pulsatile blood flow, while veins offer compliance and feature valves to prevent backflow. Capillaries, on the other hand, facilitate efficient nutrient and gas exchange between blood and tissues.

As illustrated in Fig. 1A, both arteries and veins are composed of three concentric layers: the intima, media, and adventitia [65]. The intima, the innermost layer, comprises a monolayer of ECs adhering to the internal elastic lamina. The media, positioned in the middle, is predominantly composed of SMCs and elastic fibers, providing mechanical strength to the vessel wall. The adventitia, the outermost layer, primarily consists of fibroblasts and collagen fibers, which provide structural support to the blood vessel [65].

Endothelial cells play a central role in maintaining vascular homeostasis and regulating various functions, including vascular tone, blood flow, inflammation, angiogenesis, and thrombosis [66]. Endothelial cells respond to changes in their microenvironment, such as hemodynamic forces and biochemical stimuli, by modulating their phenotype and function. Endothelial cells are integral to the regulation of blood coagulation and thrombosis by expressing procoagulant and anticoagulant factors. Under normal conditions, they maintain an antithrombotic surface by releasing anticoagulant molecules such as thrombomodulin [67]. However, in response to vascular injury or inflammation, affecting thrombus formation through distinct mechanisms. Vascular injury can damage endothelial cells, exposing subendothelial cells, the basement membrane and collagen, initiating platelet aggregation and the coagulation cascade to form a stable clot that prevents excessive blood loss [68]. Thus, any perturbations of the regulatory pathways could increase the risk of thrombus formation. Additionally, inflammation increases procoagulant factors, as well as impairs endothelial cell function, increases vascular permeability, and promotes the infiltration of inflammatory cells and platelets, further exacerbating thrombus formation [69]. These disturbances influence anticoagulant and procoagulant functions of ECs, playing a crucial role in cardiovascular diseases [70]. This multifaceted functionality underscores the significance of ECs in vascular health and disease.

Smooth muscle cells are another essential component of the vascular system, working in tandem with ECs to maintain vascular function. While endothelial cells primarily regulate vascular tone and blood flow, SMCs contribute to the structural integrity and contractility of blood vessels [71]. These cells are responsible for the constriction and dilation of arteries and veins, thereby controlling blood pressure and ensuring efficient blood circulation throughout the body. The regulation of vascular tone involves a multitude of intricate pathways, which are both numerous and complex, including signaling transduction mechanisms governing the contractile machinery [72], as well as the modalities controlling dynamic changes in cytosolic Ca^2+^ concentrations, which play a crucial role in determining the contractile state [73,74]. The primary player in signaling and mechanics governing SMC contraction and relaxation is the 20 kDa myosin light chain protein (MLC_20_). When activated, MLC_20_ triggers the Mg^2+^ ATPase activity of myosin, facilitating its binding to actin filaments and subsequent sliding, ultimately leading to cell and muscle contraction [75]. Within the cell, calcium ions (Ca^2+^) bind with calmodulin, triggering the activation of myosin light chain kinase (MLCK) and thereby instigating SMC contraction. Similar to ECs, SMCs also respond to various physiological and pathological stimuli, adjusting their contractile activity accordingly. Moreover, SMCs play a crucial role in vascular remodeling, a process essential for adapting blood vessel structure to changing physiological demands or in response to injury [19].

Given the structural diversity and the critical roles of vascular cells in thrombosis, it is essential to incorporate these features in bioprinted models. By recapitulating the multilayered structure of blood vessels and the presence of key cell types, such as ECs and SMCs, bioprinted models can provide a more physiologically relevant platform for studying the mechanisms of thrombosis and developing targeted therapies.

### 3.2 Mechanical characteristics of blood vessels

In addition to their structural diversity, blood vessels exhibit unique mechanical characteristics that are crucial for their proper function. The mechanical behaviors of blood vessels are determined by three types of stress (Fig. 1B): shear stress, circumferential stress and axial stress [49].

Shear stress, the frictional force exerted by blood flow on the vessel wall, plays a significant role in regulating endothelial cell function and vascular homeostasis [49,76]. Wall shear stress (*τ*_w_) measured in dynes (1 dyn/cm^2^ = 0.1 N/m^2^), is determined by factors like flow rate (*Q*), blood viscosity (*η*), and the radius of the lumen (*r*). Notably, the magnitude and pattern of shear stress can vary depending on the type of blood vessel and the local hemodynamic conditions. Generally, arteries experience higher shear stresses compared to veins. The wall shear stresses can range from 10 to 70 dyn/cm^2^ in larger arteries, such as the aorta and carotid arteries, while the values for veins are considerably lower, ranging from 1 to 6 dyn/cm^2^ [77]. However, when shear stress is disrupted or altered, it can lead to endothelial dysfunction, characterized by changes in endothelial cell morphology, permeability, and expression of procoagulant and anticoagulant molecules [78]. Additionally, abnormal shear stress patterns, such as low shear stress or disturbed flow, can promote platelet adhesion, activation, and aggregation on the vessel wall, initiating thrombus formation [79].

Circumferential stress and axial stress are also important mechanical factors that influence vascular function and stability. Circumferential stress (*τ*_c_) is the force exerted tangentially on the vessel wall, while axial stress (*τ*_a_) is the force applied along the longitudinal axis of the vessel [49]. These stresses are primarily governed by blood pressure and contribute to the mechanical behavior of blood vessels.

To withstand the complex mechanical forces exerted by blood flow and maintain their structural integrity, blood vessels exhibit unique mechanical properties, such as nonlinear elasticity, viscoelasticity, and anisotropy [80]. The nonlinear stress-strain relationship of blood vessel walls is attributed to the presence of collagen and elastin fibers, which have different mechanical properties and contribute to the overall mechanical behavior of the vessel wall. Viscoelasticity, the time-dependent response to deformation, arises from the complex interactions between the cells and extracellular matrix components. The anisotropic nature of blood vessel walls, resulting from the orientation and distribution of collagen and elastin fibers, leads to directional-dependent mechanical properties [80]. Lastly, to prevent rupture or permanent deformation, vascular conduits need to possess sufficient strength. Therefore, burst pressure (the maximum pressure endured by the vessel before rupture) becomes one of the most critical parameters [81]. Typically, burst pressure is determined by applying pressure at a rate of 80–120 mmHg/s to the vascular conduit to measure the internal pressure it can withstand before rupturing [81].

Taken together, incorporating these mechanical characteristics in the next generation biomimetic vascular models is crucial for accurately recapitulating the biomechanical environment of native blood vessels. By using advanced biomaterials with tunable mechanical properties and incorporating key structural features, such as aligned fibers, the next generation biomimetic models should better mimic the mechanical behavior of blood vessels. Moreover, integrating vascular constructs with microfluidic systems allows for the precise control of hemodynamic conditions, enabling the investigation of the effects of shear stress and other mechanical forces on thrombosis.

## 4 Bioprinting techniques generate the next generation vascular models

Bioprinting has emerged as a powerful tool for fabricating the next generation of biomimetic vascular models that closely recapitulate the structural and mechanical complexity of native blood vessels [82]. By enabling precise control over the spatial arrangement of cells, ECM components, and biomaterials in a 3D environment, bioprinting offers unprecedented opportunities for creating advanced vascular constructs for thrombosis research [[83], [84], [85]]. In this section, we will discuss the key aspects of the bioprinting process, including the preparation stage, current bioprinting techniques, and their applications in generating biomimetic vascular models.

### 4.1 Preparation stage: imaging, design and bioink selection

**Fig. 3:**
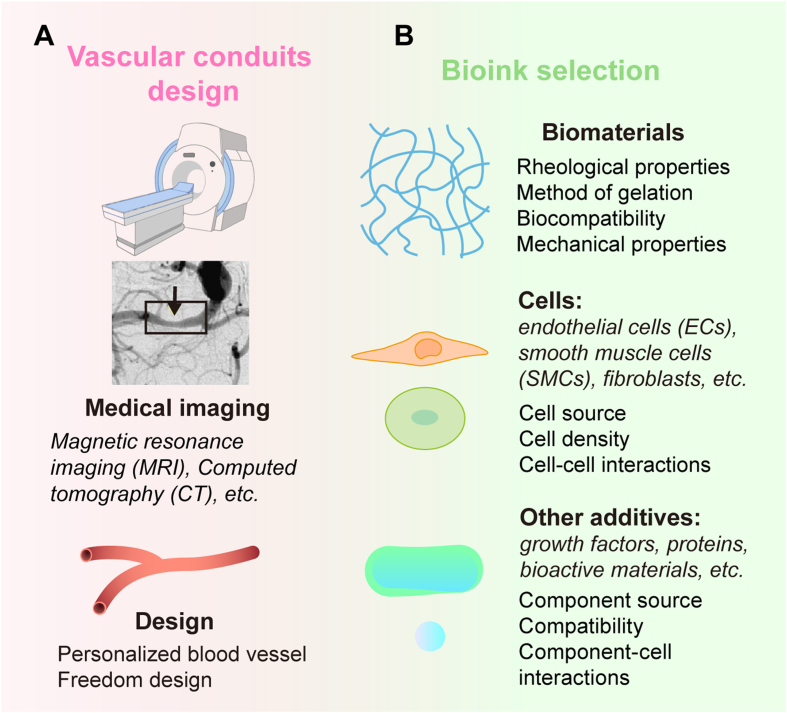
**Preparation stages before bioprinting vascular conduits. (A)** Acquisition of anatomical data. By using MRI, CT, and other medical imaging techniques detailed information on the structure, dimensions, and spatial arrangement of vascular networks and cells could be obtained. This is crucial for the design of bioprinted patient-specific vascular conduits. **(B)** Selection of bioinks with suitable characteristics for bioprinting. Bioinks are often composed of hydrogels, which need to possess appropriate rheological and mechanical properties to ensure successful bioprinting. The choice of cells is equally important; for instance, ECs, SMCs and fibroblasts can be selected to accurately replicate the structure and function of natural blood vessels. Additionally, bioactive components of the bioinks are carefully considered to ensure printability, structural integrity, and support for cell growth and function.

The quality, functionality, and suitability of the final bioprinted product, are directly influenced the preparation stage. Before commencing the bioprinting process, several crucial steps are undertaken to prepare for the fabrication of vascular conduits. These include imaging, design, and the selection of appropriate bioink. As per the definition of the International Society of Biofabrication, bioink is *‘a formulation of cells suitable for processing by an automated biofabrication technology that may also contain biologically active components and biomaterials'* (Fig. 3) [86].

First and foremost, clinical imaging modalities, such as Magnetic Resonance Imaging (MRI), Computed Tomography (CT), Optical Coherence Tomography (OCT), Ultrasound (US) and X-ray angiography, are adopted to capture the detailed anatomical structures of blood vessels [[87], [88], [89]]. These imaging modalities provide high-resolution data on the architecture, dimensions, and spatial arrangement of vascular networks, enabling the creation of patient-specific vascular models. Table 2 summarizes the key features and capabilities of these imaging techniques in the context of vascular imaging. While MRI and CT provide precise depictions of vascular shape and structure, they require long scan time and incur higher costs [88,90]; OCT offers high-resolution optical imaging, particularly useful for observing small vessels but is limited to shallow tissue depths [89,91]; Ultrasound is portable and non-invasive, suitable for real-time observation and tracking [90,92,93]; X-ray imaging offers vascular shape information but entails higher radiation doses limiting prolonged observation [90,94].

**Table 2:** **Comparison of different vascular imaging techniques.** The table summarizes the key features of various imaging modalities used for capturing the geometry and structure of blood vessels, including MRI, CT, OCT, US, and X-ray angiography. Each technique is evaluated based on its resolution, ability to capture vessel geometry, and typical image processing steps. This information is crucial for selecting the appropriate imaging modality for the preparation stage, ensuring accurate reconstruction of vascular geometry for the design of bioprinted vascular conduits.

Imaging Modality	Resolution	Geometry Capture Ability	Vascular Image Processing	Ref.
Magnetic Resonance Imaging (MRI)	＜1 mm	Offers detailed images of vessel wall and surrounding structures, especially smaller vessels, without ionizing radiation.	1. Preprocessing: involves denoising and enhancing contrast to improve image quality. 2. 3D reconstruction: constructs a 3D model from 2D image slices. 3. Segmentation and analysis: analyzes the 3D model to create geometric representations of vessels.	[88,90,95]
Computed Tomography (CT)	＜1 mm	Offers detailed cross-sectional images of vessels, particularly suitable for large vessels and detecting abnormalities.	1. Image acquisition: capture detailed cross-sectional images. 2. 3D reconstruction: combines multiple cross-sectional images to form a 3D model. 3. Segmentation and analysis: analyzes the 3D model to create geometric representations of vessels.	[88,90,96]
Optical Coherence Tomography (OCT)	10 μm	Provides high-resolution images of vessel walls, useful for assessing microvessels and plaque buildup.	1. Image acquisition: captures high-resolution images. 2. Processing: enhances images using specific algorithms for better visualization.	[89,91]
Ultrasound (US):B mode	0.1 mm–1 mm	Provides grayscale images of vessel walls and microscopic characteristics of the lesions	1. Image acquisition: generate grayscale images of vessel walls. 2. Processing: enhances grayscale images to improve visualization and diagnostic capability.	[90,92,93]
Ultrasound (US):Doppler	0.1 mm–1 mm	Offers real-time and prolong imaging of blood flow dynamics	1. Flow measurement: measures blood flow velocity and direction using the Doppler effect. 2. Processing: provides real-time analysis of blood flow dynamics and patterns.	[90,92,97]
X-ray angiography	100 μm	Provides detailed vessel images, useful for identifying blockages or abnormalities.	1. Image acquisition: captures detailed vessel images. 2. Processing: enhances contrast to highlight details of vessels and detect abnormalities.	[90,94]

Subsequently, 3D models of the vascular network are reconstructed using image processing software to provide precise geometric data for bioprinting design. During the design phase, based on the imaging data, the desired vascular conduit structure is designed using computer-aided design (CAD) software [98]. Factors such as vessel diameter, branching patterns, and overall geometry are considered to simulate physiological conditions. Simultaneously, it is ensured that the design allows for proper nutrient and oxygen diffusion to support cell viability within the bioprinted structure. These specific models can be used to study the formation of blood clots under controlled conditions, providing insights into the interplay between vascular geometry, flow dynamics, and clot formation, as well as the underlying mechanisms of thrombosis [99].

Finally, during bioink selection, biomaterials compatible with the selected bioprinting technology and capable of supporting cell growth and function are chosen. Bioinks are often composed of hydrogels (e.g., alginate [[100], [101], [102]], gelatin [103,104], gelatin methacryloyl (GelMA) [105], collagen [106,107], hyaluronic acid (HA) [108,109], and polyethylene glycol (PEG) [110,111]), and are selected to mimic the native ECM of blood vessels. Additionally, the rheological properties of biomaterials, including viscosity, shear-thinning behavior, and gelation kinetics, are considered to ensure printability and structural integrity [[112], [113], [114]]. More importantly, bioactive components such as growth factors or signaling molecules are incorporated into the bioink formulation to promote cell adhesion, proliferation, and differentiation within the bioprinted structure [115]. Moreover, the mechanical properties of the bioink are evaluated to match the target tissue stiffness and provide appropriate biomechanical cues for cell behavior. By carefully addressing these aspects in the preparation stage, the fabrication process of vascular conduits for thrombosis research can be optimized, leading to more physiologically relevant and functional models for exploring thrombosis mechanisms and developing therapeutic interventions.

### 4.2 Current bioprinting techniques

In the current landscape of bioprinting techniques, various modalities have been used to construct vascular conduits, including inkjet-based, lithography-based and extrusion-based bioprinting techniques. Each of these techniques are fundamentally different in terms of technological operations and possess distinct strengths and weaknesses. The principles, applications, recent advancements associated with each method will be comprehensively explained., Also, their advantages and disadvantages in terms of cell density, cell viability, resolution and multi-material printing capability were summarized in Table 3.

**Table 3:** **Comparison of different bioprinting techniques in terms of cell density, cell viability, resolution, and material properties.** Inkjet-based bioprinting offers high resolution and minimal impact on cell viability but requires low cell densities and bioink viscosities. Extrusion-based bioprinting accommodates higher cell densities and a broader range of biomaterial viscosities but has lower resolution and cell viability. Lithography-based bioprinting achieves high resolution and cell viability, with the ability to print multi-material constructs, but has limitations in terms of cell density.

Bioprinting Technique	Cell Density	Cell Viability	Resolution	Material	Reference
Inkjet-based bioprinting	＜10^6^ cells/mL	>85 %	＜50–75 μm	3.5–12 mPa·s	[116,117]
Extrusion-based bioprinting	＞10^8^ cells/mL	40 %–80 %	>100 μm	30 mPa s - 6 × 10^7^ mPa·s	[[117], [118], [119], [120]]
Lithography-based bioprinting	＜10^8^ cells/mL	>85 %	＜5–10 μm	Multi-material	[[117], [118], [119]]

#### 4.2.1 Inkjet-based bioprinting

Inkjet-based bioprinting utilizes thermal or piezoelectric inkjet printheads to dispense bioinks onto a substrate in a controlled manner [121]. Thermal inkjet bioprinting uses heat to create vapor bubbles that propel droplets of bioink onto the substrate, with minimal impact on cell viability due to instant heating, while piezoelectric bioprinting utilize electrically induced vibrations for droplets ejection [121]. Although it offers high resolution and minimal impact on cell viability, the cell density needs to be maintained below 10^6^ cells/mL to mitigate shear stress that could potentially rupture the cell membrane during the printing process [116]. The viscosity of bioink, typically ranging from 3.5 to 12 mPa·s, undoubtedly influences the selection of suitable biomaterials, while also posing potential nozzle clogging issues, particularly with high cell density or viscous materials [116].

Despite these challenges, inkjet-based bioprinting has been instrumental in the fabrication of complex tissue constructs with intricate features. Alginate and CaCl_2_ are widely employed to create tubular structures by forming alginic acid nanoparticles in CaCl_2_ solutions, which are subsequently assembled [101,102]. Kesari et al. [101] pioneered the bioprinting of tubular blood vessels by inkjet printing the CaCl_2_ solution into an alginate bath. Building upon this work, Nakamura et al. [102] reversed the process by ejecting alginate droplets into CaCl_2_ solution to fabricate tubular structures with 200 μm channels. However, due to the instability of droplet stacking and difficulty in controlling the structure, free-form shapes are able to be fabricated when sufficient support material is used, where the constructs are still limited to thin structures with only a few layers [122]. This can result in poor mechanical properties and may also lead to the absence or incomplete formation of vessel structures.

**Fig. 4:**
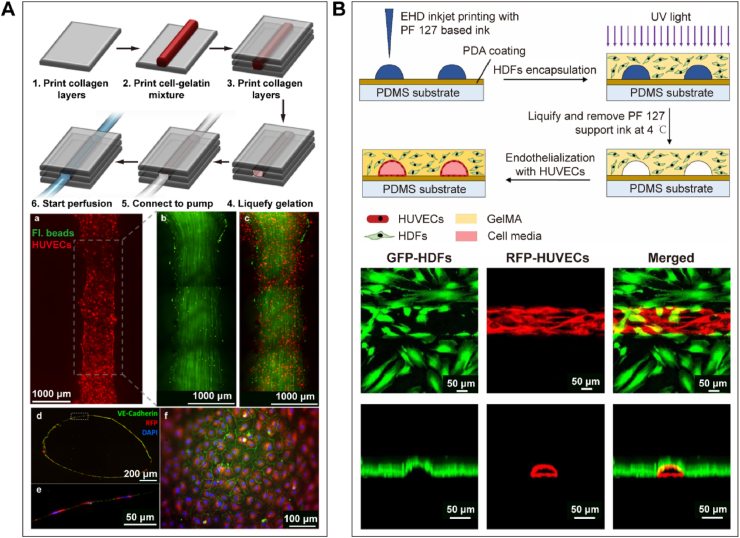
**Inkjet-based bioprinting for the fabrication of vascular structures.** (**A**) Lee et al. constructed functional vascular channels using a cell-gelatin mixture and a layer-by-layer inkjet printing approach. The channels had widths ranging from 0.7 to 1.5 mm and heights from 0.5 to 1.2 mm, with a single layer of HUVECs incorporated along the inner surface of the channels [123]. (**B**) Zheng et al. employed EHD inkjet bioprinting to fabricate microvascular structures with functional endothelial layers, featuring characteristic dimensions as small as 30 μm [124].

However, Lee et al. [123] created a perfusable functional *in vitro* vascular channel within a collagen matrix using a layer-by-layer inkjet-based printing approach. As shown in Fig. 4A, a single layer of human umbilical vein endothelial cells (HUVECs) formed along the inner surface, with channel widths ranging from 0.7 to 1.5 mm and heights from 0.5 to 1.2 mm. Moreover, the vascular structure exhibited barrier function against plasma proteins and dextran molecules [123].

In addition, electrohydrodynamic (EHD) inkjet bioprinting uses an electric field to induce fluid flow, resulting in smaller droplets and improved resolution [125]. Zheng et al. [124] employed this technique to fabricate microvascular structures with feature sizes as low as 30 μm. They utilized Pluronic F127 as sacrificial templates and GelMA containing human dermal fibroblasts (HDFs) as the permanent matrix. After removing Pluronic F127, endothelialization of the inner channel was achieved, leading to the formation of a functional endothelial layer (Fig. 4B) [124].

#### 4.2.2 Extrusion-based bioprinting

In contrast to inkjet-based bioprinting, extrusion-based bioprinting stands out for its versatility and suitability for printing large-scale structures. By extruding bioinks through a nozzle under pneumatic or mechanical pressure, this technique accommodates a broader range of biomaterial viscosities, ranging from 6 to 30 × 10^7^ mPa·s and facilitates the incorporation of diverse cell types and growth factors [120]. However, it is typically limited to resolutions greater than 100 μm after encapsulating cells, due to factors like nozzle diameter and gelation kinetics [120].

Direct extrusion printing is also heavily dependent on the shear-thinning properties of high-viscosity inks, where external forces are required to facilitate polymer chain rearrangement to obtain optimal flow properties. However, optimizing the formulation of bio-inks to achieve a balance between resolution and cell viability remains a challenge [126]. This method requires materials to have sufficient rigidity to support the layer-by-layer accumulation of bioink, thus limiting the types of bioinks and the final achievable size range.

**Fig. 5:**
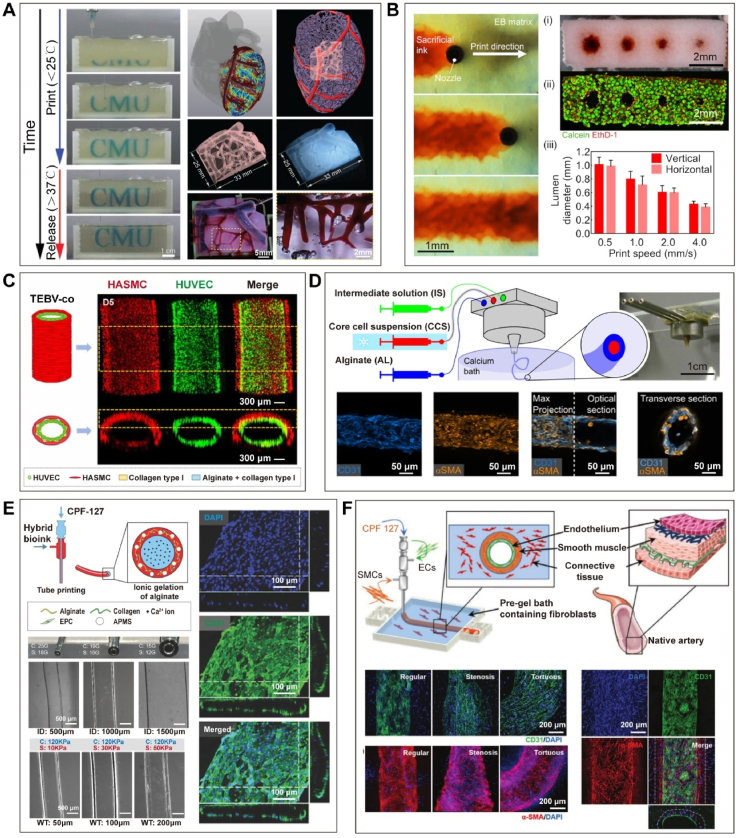
**Extrusion-based bioprinting for the fabrication of vascular structures. (A)** The FRESH technique was used to construct a multi-scale vascular network within a human heart model, demonstrating interconnected blood vessels with diameters of approximately 100 μm [107]. **(B)** The SWIFT method was employed to construct tubular structures with diameters ranging from 400 μm to 1 mm by controlling the printing speed [128]. **(C)** E Bosch Rúe et al. encapsulated HUVECs and HASMCs separately in collagen and alginate to produce double-layered hollow fibers with parallel and perpendicular cellular arrangements, respectively [129]. **(D)** Andrique et al. utilized alginate as the outer layer and a core cell suspension containing SMCs and ECs as the inner layer, facilitating the formation of a double-layered structure through the directed self-assembly of SMCs. With the outer diameter about 448 ± 12 μm, while the inner diameter about 321 ± 21 μm [130]. **(E)** Gao et al. encapsulated EPCs in a hybrid bioink composed of alginate and dECM, enabling the printing of vascular conduits with various inner diameters and wall thicknesses [131]. **(F)** Using alginate and ECM-based bioinks, endothelial and smooth muscle layers were encapsulated to print mimics of regular arteries, stenotic arteries, and tortuous arteries [132].

Additionally, indirect bioprinting methods, by removing temporary or sacrificial materials to form vascular structures, provide an alternative strategy for fabricating vascularized tissue constructs, allowing for higher resolution and the construction of smaller diameter conduits. The Freeform Reversible Embedding of Suspended Hydrogels (FRESH) technique, designed by Feinberg et al. [107], allow bioinks to be printed within a soluble gelatin support bath. As shown in Fig. 5A, this approach achieves a resolution as small as 20 μm for cell-free printing and enables the fabrication of perfusable multiscale vascular systems, with diameters smaller than 100 μm remaining patent [107]. However, the resolution remains relatively low when cells are added, approximately 10 times the diameter of the cells, and prolonged printing times can damage the cells [127]. Lewis et al. [128] embedded sacrificial ink in a temperature-responsive ECM support bath to manufacture embedded vascular channels through the removal of the template. In their study, embedded tubular structures with diameters ranging from 400 μm to 1 mm (Fig. 5B) can be constructed by using a nozzle with a diameter of 250 μm, a constant volume flow rate and varying the printing speed [128]. Despite these advancements, residual material remnants within conduits post-removal of sacrificial materials remain a concern as it may affect hemodynamics as well as the pattern and rate of thrombus formation. In addition, achieving the printing of multiple concentric layers composed of several different cell types and hydrogels using the above-mentioned extrusion printing methods is challenging.

Recent technological advancements in coaxial bioprinting, a variation of extrusion-based printing first performed in 2011, have been utilized to enhance resolution and accuracy in creating blood vessels within intricate constructs [133]. It can simultaneously deliver bioink and cross-linking agents as separate flow streams through a concentric nozzle, allowing for the single-step generation of hollow, standalone vascular conduits [134]. By employing different nozzle setups and bioink designs, researchers have made notable contributions to this field. In the study of E Bosch Ru'e et al. [129], HUVEC and human arterial smooth muscle cell (HASMC) were encapsulated separately in collagen and alginate, then were extruded through a triple-coaxial nozzle along with sacrificial polymer to form double-layered hollow fibres (Fig. 5C). The diameter of bioprinted blood vessels is related to the injection speed; increasing the speed causes the outer diameter to change from approximately 1500 to 1700 μm, while the inner diameter changes from 1300 to 1500 μm. Notably, HUVEC and HASMC exhibited parallel and perpendicular arrangements resembling *in vivo* organization, respectively. The resulting structure was robust, capable of withstanding infusion rates of 25 and 50 ml/h [129]. Andrique et al. [130] designed a dual-layer, perfusable vascular conduit with an outer diameter of approximately 448 ± 12 μm and an inner diameter of approximately 321 ± 21 μm. Additionally, this conduit exhibited adjustable contractility when stimulated. Alginate solution (AL) was used as the outer layer and core cell suspension (CCS) containing ECM as the inner layer, directed self-assembly of SMCs and ECs allows for the formation of a dual-layered structure within one day (Fig. 5D) [130]. Moreover, Gao et al. produced a series of notable publications from 2017 to 2023 on the use of coaxial printing for different applications. In their 2017 work, endothelial progenitor cells (EPCs) and antiplatelet microparticles (APMs) are encapsulated in a mixed bioink consisting of alginate and decellularized extracellular matrix (dECM) for printing, and ultimately results in an interacting monolayer endothelium (Fig. 5E) [131]. By using nozzles of different sizes, the inner diameter can be adjusted within the range of 500–1500 μm. Additionally, by controlling the flow rate, the wall thickness can be adjusted within the range of 50–200 μm [131]. Over the following years, they refined their work by utilizing cell-laden alginate and ECM-based bioinks to create tunable vascular equivalents containing endothelial and smooth muscle layers [135,132]. Based on the dual-layer tube with an inner diameter of 600 μm, an inner wall thickness of 50 μm, and an outer wall thickness of 200 μm, they successfully printed regular, stenotic (with sizes reduced to 325, 35, and 140 μm in the narrowed region), and tortuous artery mimics. Demonstrating their potential in vascular functionality (including selective permeability, antiplatelet/leukocyte adhesion, angiogenesis, inflammatory pathways, and vascular remodeling under shear stress) and the response of endothelial cell dysfunction to atherosclerosis (Fig. 5F) [132]. However, achieving microvessel resolution with extrusion-based bioprinting still remains challenging, so the utilization of additional printing techniques beyond extrusion becomes essential.

#### 4.2.3 Lithography-based bioprinting

Compared to inkjet or extrusion-based bioprinting, lithography-based bioprinting can fabricate tissue structures with greater precision and higher resolution (＜5–10 μm), and it is more suitable for sensitive cell types such as stem cells [118,136,137].

**Fig. 6:**
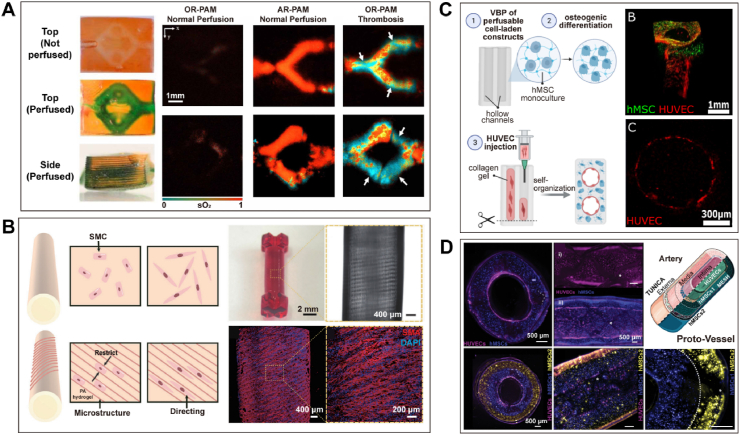
**Lithography-based bioprinting for the fabrication of vascular structures. (A)** DLP-based bioprinting was used to print vascular structures, with thrombus detection conducted through the measurement of hemoglobin oxygen saturation (sO_2_) [138]. **(B)** Yang et al. designed spiral grooves spaced at approximately 50 μm intervals to guide the direction of smooth muscle cell growth on a PEGDA-acrylamide (PA) hydrogel [110]. **(C)** Ultrafast scanning volumetric bioprinting (VBP) was employed to print a soft GelMA construct containing hMSCs, with subsequent seeding of HUVECs to form a perfusable structure [139]. **(D)** By combining volumetric printing and melt electrowriting, tubular structures were fabricated with three layers containing HUVECs, hMSCs_1_, and hMSCs_2_, respectively [140].

Digital light processing (DLP)-based bioprinting, with its superior micrometer-level printing resolution and ability to rapidly solidify entire monomer layers at once, is gaining attention for its significant potential in constructing vascular and thrombosis models [138]. Ma et al. [138] successfully bioprinted an *in vitro* vascular model with internal microchannels having a depth of 1.7 mm and a resolution of 200–350 μm. Subsequently, thrombus detection was conducted by measuring hemoglobin oxygen saturation (sO_2_) (Fig. 6A). Notably, to address the shallow imaging depth limitation of traditional imaging devices, the study utilized photoacoustic microscopy (PAM) for visualization at depths reaching up to 3.6 mm [138]. However, the resulting 3D-printed structures are often quite fragile, which poses challenges, particularly when producing vessels that need to withstand high pressure and bending. Yang et al. [110] utilized DLP-based bioprinting to fabricate hydrogel structures with a resolution of approximately 30 μm, and then they printed spiral grooves spaced at approximately 50 μm intervals to guide the direction of SMCs growth seeded on the PEGDA-Aam (PA) hydrogel: acrylamide, PEGDA, and lithium phenyl-2,4,6-trimethylbenzoylphosphinate (LAP) were respectively used as the monomer, crosslinker, and photoinitiator (Fig. 6B). The PA hydrogel used in this study exhibited adjustable elastic modulus in the range of tens to hundreds of kPa and demonstrated excellent structural stability [110].

Moreover, volumetric bioprinting is a more efficient method for constructing intricate structures with excellent smooth surface finishes at high printing speeds, consequently maintaining high cell viability [141]. It innovatively employs cross-projection and photopolymerization techniques by rotating the bioink container [142]. Jenny et al. [139] utilized ultrafast scanning volumetric bioprinting (VBP) technology to print an injectable *in vitro* model with hollow channels smaller than 1 mm. This model contained hMSCs using soft GelMA (<5 kPa), which was subsequently seeded with HUVECs on the surface (Fig. 6C). Gabriel et al. [140] employed volumetric printing to create a GelMA layer encapsulating hMSCs and a melt electrowriting (MEW) scaffold to provide mechanical stability to the structure, and seeded HUVECs internally (Fig. 6D). Furthermore, by adding a second volumetric printing step with a suspension of new cell types, they successfully fabricated a three-layered structure resembling natural vessels with a layered architecture [140]. The combination of volumetric bioprinting with melt electrowriting creates structures that are more robust and durable.

### 4.3 Advances in bioprinting mechanically and functionally relevant vascular conduits

Despite recent progress in bioprinting, these bioprinted vascular conduits still only partially recapitulate the structure and function of native blood vessels. They generally display weaker mechanical strengths in comparison to their native counterparts and, subsequently, have limited biological applications under physiological environments [143]. The size and architecture of the resulting blood vessel are also constrained by the dimensions of the coaxial nozzle. There is an existing need for a technology capable of independently bioprinting each layer within the same construct, accomplishing this task swiftly and efficiently.

**Fig. 7:**
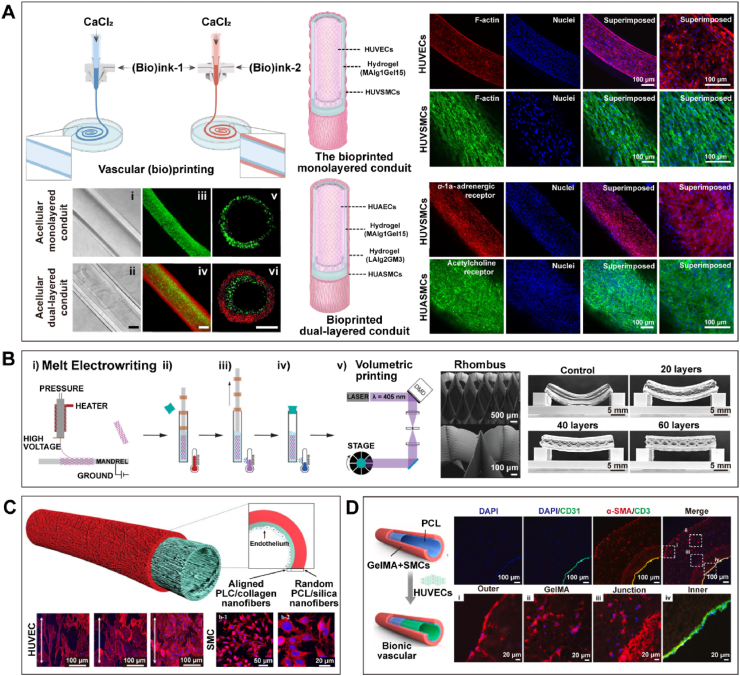
**Advances in bioprinting mechanically and functionally relevant vascular conduits. (A**) The DN hydrogel bioink system enables the bioprinting of vascular conduits containing endothelialized intima and functionally enhanced muscle layers in both veins and arteries [143]. **(B)** Building upon the foundation of melt electrowritten scaffolds, volumetric bioprinting is employed to construct vascular conduits with excellent mechanical properties [140]. **(C)** Electrospinning was employed to create a double-layered small-diameter blood vessel, where the inner fibers were longitudinally aligned while the outer fibers were randomly arranged [146]. **(D)** Extrusion-based bioprinting was combined with electrospinning to construct a double-layered tubular structure [147].

Stronger vascular conduits have been reported using synthetic polymer-based bioinks to create nanocomposite hydrogels and double-network (DN) hydrogels with greater toughness [144,145]. Although mechanically strong and cytocompatible, these materials have limited biofunctionality in their inability to support the spreading and proliferation of embedded cells. This challenge of bioprinting mechanically and functionally relevant vascular conduits was addressed in the recent work by Wang et al. in 2022 [143]. They presented a stretchable DN hydrogel bioink system for the bioprinting of small - diameter venous conduits that favorably recapitulates structural and biological functions and is functionalized with endothelial and muscular layers that make up the tunica intima and tunica media, respectively (Fig. 7A) [143]. The DN hydrogel exhibited higher strength and stretchability, with a tensile strength of 197.7 kPa, Young's modulus of 142.8 kPa, tensile strain of 207.3 %, and superior hysteresis ratio that indicates stress-transfer and additional energy dissipation, as well as a slightly higher burst pressure (1113.1 mmHg) compared to mouse vena cava (297.1 mmHg) [143]. The authors concluded that these bioprinted venous conduits possess superior mechanical and physiological properties that mimic important features of native veins, with strong potential for *in vitro*, *ex vivo*, and *in vivo* applications in the future. Building upon and integrating the work of Wang et al. with the field of microfluidics for the study of venous thrombosis would yield an *in vitro* model with greater biomimicry to native vasculature not yet seen before. Combining these two fields and applying them to study relevant blood clotting diseases is a novel driving motivation behind this body of work.

In addition, Gabriel et al. [140] combined volumetric bioprinting with melt electrowriting to create structures that are more robust and durable (Fig. 7B). The elongation at break of the melt electrowritten scaffold made from medical-grade polycaprolactone (PCL) reaches 166 %, surpassing the strain levels of physiological blood vessels. Specifically, when presenting a 70° rhomboid structure, the peak stress reached 73.0 ± 21.5 kPa, the Young's modulus reached 10.8 ± 3.3 kPa, and the burst pressure reached 1.58 ± 0.17 bar. Moreover, as the layer height increased, the bending resistance also increased [140]. This kind of method addressed the challenge posed by the fragility of 3D printed structures resulting from the use of cell-friendly hydrogels, and it is particularly crucial when producing vessels that need to withstand high pressure and bending.

Electrospinning shows great potential in fabricating small - diameter blood vessels [148]. Park et al. [146] fabricated a double-layered tubular structure with a diameter of 3 mm using electrospinning, with the inner layer composed of poly (ε - caprolactone) (PCL) and collagen nanofibers aligned longitudinally, while the outer layer consisted of randomly arranged PCL and silicon dioxide hybrid nanofibers (Fig. 7C). The inner layer facilitated rapid endothelialization of the luminal surface, while the outer layer provided excellent mechanical strength and supported fibroblast growth, with longitudinal ultimate tensile strength (UTS) around 3.5 MPa and Young's modulus around 4.5 MPa, and circumferential UTS around 3 MPa and Young's modulus around 3.5 MPa. The fracture elongation rates (in the longitudinal and circumferential directions, respectively) were approximately 69 % and 70 %, comparable to the elongation rates of natural coronary arteries (45–99 %) [146]. In the study of Jin et al. [147], a combination of extrusion-based bioprinting and electrospinning was employed to create a dual-layered tubular structure (Fig. 7D). The outer layer, containing SMCs, was printed using GelMA, while the inner layer was electrospun with PCL. Subsequently, the structure was perfused with HUVECs. The resulting tubular structure had an inner diameter of 1.78 mm and a burst pressure of up to 2035 ± 173.8 mmHg [147]. These advancements hold significant potential for biomedical applications, offering improved biomimicry and functionality for *in vitro* studies.

## 5 Bioprinting vascular conduits for thrombosis studies

The advancements in bioprinting techniques have paved the way for the development of biomimetic vascular conduits that closely recapitulate the structural and mechanical properties of native blood vessels. These bioprinted vascular models have emerged as powerful tools for studying the complex mechanisms of thrombosis and developing novel therapeutic strategies. In this section, we will discuss the key considerations for designing bioprinted vascular conduits for thrombosis studies. Integrating bioprinted vascular models with microfluidic systems can replicate hemodynamic conditions, offering more realistic models for thrombosis research. By incorporating patient-specific data, the clinical relevance of printed models is enhanced, enabling personalized disease studies and therapeutic strategies.

### 5.1 Incorporating key structural and mechanical features in bioprinted thrombosis models

To create accurate and physiologically relevant thrombosis models, it is crucial to incorporate the key structural and mechanical features of native blood vessels in bioprinted constructs. This includes replicating the multilayered structure of the vessel wall, comprising the intima, media, and adventitia, as well as the presence of ECs, SMCs, and ECM components such as collagen and elastin [14]. More importantly, a functional endothelial cell layer lining the inner surface of the vessel is necessary for modeling vascular physiology and thrombosis initiation, which could regulate coagulation and inflammation [149]. By recapitulating these structural elements, bioprinted models can better mimic the complex cell-cell and cell-matrix interactions that play a significant role in the pathophysiology of thrombosis.

Bioprinted vascular models should aim to mimic this complex architecture by incorporating the relevant cell types and ECM components in a spatially controlled manner. This can be achieved through the use of multi-material bioprinting techniques, such as coaxial bioprinting or multi-nozzle extrusion bioprinting, which allow for the precise deposition of different bioinks in a layer-by-layer fashion [133,134]. By carefully selecting the appropriate biomaterials and cell sources, researchers can create bioprinted constructs that closely resemble the cellular composition and organization of native blood vessels.

In addition to the structural features, incorporating the mechanical properties of blood vessels is crucial for developing functional thrombosis models. Native blood vessels exhibit unique mechanical characteristics, such as nonlinear elasticity, viscoelasticity, and anisotropy, which arise from the complex interactions between cells and ECM components [80]. These mechanical properties play a significant role in regulating vascular function and influencing the hemodynamic environment, which in turn affects the process of thrombosis [80].

To recapitulate these mechanical properties, bioprinted vascular conduits should be fabricated using biomaterials with tunable mechanical characteristics. For example, the use of hydrogels with adjustable crosslinking densities or the incorporation of reinforcing materials, such as electrospun fibers or 3D printed scaffolds, can help to achieve the desired mechanical strength and elasticity [150]. Moreover, the alignment of cells and ECM components within the bioprinted constructs can be controlled to mimic the anisotropic nature of native blood vessels, which is essential for maintaining their mechanical integrity and function.

Another important aspect of designing bioprinted thrombosis models is the incorporation of relevant hemodynamic conditions. Blood flow and shear stress are critical factors that influence the initiation and progression of thrombosis [[49], [50], [51]]. To study the effects of hemodynamics on thrombosis, bioprinted vascular conduits can be integrated with microfluidic systems that allow for the precise control of flow rates, shear stresses, and pulsatility [11,151]. By subjecting the bioprinted constructs to physiologically relevant hemodynamic conditions, researchers can investigate the complex interplay between blood flow, vascular cells, and thrombosis in a more realistic setting.

### 5.2 Selecting appropriate biomaterials and cell sources for functional vascular conduits

The choice of biomaterials and cell sources is a critical consideration in the development of bioprinted vascular conduits for thrombosis studies. The ideal bioinks should be biocompatible, printable, and capable of supporting the growth and function of vascular cells [[112], [113], [114]]. Table 4 provides an overview of potential hydrogels for fabricating functional vascular models through bioprinting. Notably, gelatin and Pluronic F127 stand out as the prevailing sacrificial biomaterials in current 3D bioprinting practices [152]. Gelatin methacryloyl is a derivative of gelatin through methacrylate modification, not only enhances mechanical properties but also retains excellent cell compatibility, rendering it adaptable across various bioprinting techniques [124,139,153,154]. Additionally, alginate, due to its shear-thinning properties, can reduce the impact of shear stress on cells during the printing, thereby enhancing cell viability. While alginate finds extensive utility in inkjet-based and extrusion-based bioprinting, its full potential for vascularization often necessitates blending with other biomaterials [107,143,[155], [156], [157]].

**Table 4:** **Appropriate biomaterials for fabricating functional vascular models.** The table lists various materials, their key requirements, suitable bioprinting methods, and relevant references. Gelatin, GelMA, collagen, alginate, fibrin, Pluronic F127, and PEG are highlighted as promising biomaterials for creating bioprinted vascular constructs.

Material	Key Requirement Match	Suitable Bioprinting Methods	Ref.
Gelatin	Cell-adhesive (contains cell-adhesive ligands); enzymatically cleavable	Inkjet, Extrusion	[107,143,155,156]
Gelatin Methacrylate (GelMA)	Cell-adhesive (contains cell-adhesive ligands); tunable mechanical properties	Extrusion, Inkjet, Lithography	[124,139,153,154]
Collagen	Biocompatible (the main component of the ECM; providing an ideal microenvironment for cell proliferation and migration)	Inkjet, Extrusion	[107,123,158]
Alginate	Shear-thinning; short crosslinking time	Inkjet, Extrusion	[143,158,159]
Fibrin	Cell-adhesive; encouraging vascularization	Inkjet, Extrusion	[160,161]
Pluronic F127	High print resolution; special temperature sensitive	Extrusion, Inkjet	[124,154,162]
Polyethylene Glycol (PEG)	High hydrophilicity; low immunogenicity; high strength	Extrusion, Inkjet	[104,111,163]

Regarding cell sources, the incorporation of primary vascular cells, such as ECs and SMCs, is essential for recapitulating the cellular composition and function of native blood vessels [130,143]. Furthermore, the cellular arrangement within an ideal functional vascular structure should mimic that of natural blood vessels, characterized by longitudinally aligned ECs and circumferentially arranged SMCs [164]. Endothelial cells play a critical role in maintaining vascular homeostasis and regulating thrombosis, while SMCs provide mechanical support and contribute to vessel contractility [70]. However, the extraction techniques for autologous cells are invasive, often leading to morbidity at the donor site, and these cells exhibit limited proliferative and regenerative capabilities [165,166]. In contrast, stem cells possess robust proliferative properties, high differentiation rates, and can be sourced from various origins [167]. Therefore, the use of induced pluripotent stem cell (iPSC) - derived vascular cells can further enhance the clinical relevance and personalization to these models, potentially allowing for the study of individualized disease mechanisms and drug responses [168,169]. In summary, through careful consideration and selection of biomaterials and cell sources, bioprinted thrombosis models can accurately replicate the complexity of native vasculature, offering valuable insights into disease pathogenesis and paving the way for personalized therapeutic interventions. Table 5 summarizes the hydrogels, cell types, and vascular structure properties used in various bioprinting techniques for fabricating vascular conduits.

**Table 5:** **Hydrogels, cell types, and vascular structure properties used in various bioprinting techniques for fabricating vascular conduits.** The table summarizes the bioprinting techniques, hydrogels, cell types, additives, diameters, and key properties of the bioprinted vascular structures, along with the corresponding references. This information provides an overview of the diverse approaches and materials employed in the fabrication of functional vascular conduits using different bioprinting methods.

Bioprinting Technique	Hydrogels	Cells	Additives	Diameter	Properties	Ref.
Inkjet-based bioprinting	Fibrin	Human microvascular endothelial cells (HMVECs)	Thrombin and Ca^2+^	93 μm	Elastic modulus: 2.9 ± 0.8 MPa; UTS: 1.7 ± 0.5 MPa; Burst pressure: 2955 mm Hg; HMVEC proliferation and microvasculature formation were promoted.	[170]
	Alginate	NIH 3T3 mouse fibroblasts	No additives	3 mm	Structures with both horizontal and vertical bifurcations and cell viability is maintained above 90 % within 24 h after printing.	[159]
	Bioink: GelMA, substrate: PDMS, sacrificial templates: Pluronic F127 (PF-127)	Human dermal fibroblasts (HDFs) and HUVECs	No additives	30 μm or 60 μm	Perfusion structure with a functional endothelial layer.	[124]
Extrusion-based bioprinting	GelMA, poly(ethylene glycol) diacrylate (PEGDA)	Mixing: vascular smooth muscle cells (VSMCs); Seeding: ECs	Nanosilicates	Varying diameters	High printability is not affected by cell density, and the printed structure is able to mimic thromboinflammatory outcomes.	[111]
	Embedding medium: hydrophobically modified hydroxypropylmethyl cellulose (H-HPMC) and Pluronic F-127 (PF-127); Bioink: Gelatin/GelMA/Alginate/Collagen/Chitosan	HUVECs	Embedding medium: Polyethylene glycol 400 (PEG400); Bioink: No additives	Inner: 2.6 mm; Outer: 4 mm	Bioinks with different crosslinking methods can be printed in the same embedding medium; Elastic modulus can up to 20 kpa.	[154]
	External: Gelatin–PEG–tyramine (GPT); Internal: Gelatin	External: HDFs; Internal: HUVECs	External: No additives; Internal: H_2_O_2_	Inner: 485.5 ± 31.1 μm; Outer: 670 ± 39.1 μm	Perfusion structure, the endothelial cells inside were proliferated and the fibroblast cells migrated outwards.	[104]
Lithography-based bioprinting	GelMA	HUVECs and HDFs	Iodixanol (IDX)	250–600 μm	High-density, uniformly mixed cells were able to print.	[171]
	GelMA	Mixing: hMSCs; Seeding: HUVECs	No additives	<1 mm	Perfusion structure with a functional endothelial layer.	[139]

### 5.3 Challenges and future directions in bioprinted models of thrombosis

Despite the significant progress in bioprinting vascular conduits for thrombosis research, several challenges remain to be addressed. One of the main challenges is the need for improved biomaterials that can better recapitulate the complex ECM composition and mechanical properties of native blood vessels [172]. While current biomaterials have shown promise in supporting the growth and function of vascular cells, they often lack the structural and mechanical complexity found in native tissues. The development of advanced bioinks that incorporate multiple ECM components, such as collagen, elastin, and glycosaminoglycans, and exhibit tunable mechanical properties, is essential for creating more biomimetic vascular models [173].

Another challenge is the scalability and standardization of bioprinting processes for the fabrication of vascular conduits. The ability to create large-scale, reproducible, and consistent vascular models is crucial for their widespread adoption in thrombosis research. This requires the optimization of bioprinting parameters, such as print speed, nozzle diameter, and bioink formulation, to ensure the reliable and efficient production of vascular constructs. Moreover, the establishment of standardized protocols and quality control measures is necessary to facilitate the comparison and validation of results across different research groups and institutions.

The long-term stability and functionality of bioprinted vascular conduits is another important consideration. While current bioprinted models have shown promising results in short-term studies, their ability to maintain structural integrity, cellular functionality, and mechanical properties over extended periods remains a challenge. The development of strategies to promote the maturation and remodeling of bioprinted constructs, such as the incorporation of growth factors and dynamic culture conditions, is essential for creating vascular models that can mimic the long-term behavior of native blood vessels.

Future directions in bioprinted models of thrombosis include the integration of patient-specific data and the development of personalized vascular models [151,174]. By combining advanced imaging techniques, such as MRI and CT, with bioprinting technologies, researchers can create vascular conduits that accurately replicate the unique anatomical and pathological features of individual patients. This approach has the potential to revolutionize the field of thrombosis research by enabling the development of personalized diagnostic and therapeutic strategies.

Moreover, the incorporation of real-time monitoring and sensing technologies into bioprinted vascular models can provide valuable insights into the dynamic processes of thrombosis. The integration of biosensors and imaging modalities, such as optical coherence tomography and confocal microscopy, can enable the non-invasive and continuous monitoring of cellular behavior, ECM remodeling, and thrombus formation within bioprinted constructs. This real-time data can help to elucidate the complex mechanisms underlying thrombosis and guide the development of targeted interventions.

Another promising avenue for future research is the combination of bioprinted vascular models with microfluidic technologies to create advanced *in vitro* platforms for thrombosis studies. The integration of bioprinted vascular conduits with microfluidic devices can enable the precise control of hemodynamic conditions, such as shear stress and pulsatility, and allow for the real-time monitoring of cellular responses and thrombus formation. These advanced *in vitro* models can serve as powerful tools for screening novel antithrombotic therapies and investigating the effects of hemodynamics on thrombosis in a high-throughput and cost-effective manner.

In conclusion, bioprinting vascular conduits for thrombosis studies hold immense potential for advancing our understanding of the complex mechanisms underlying thrombosis and developing novel therapeutic strategies. By incorporating key structural and mechanical features, selecting appropriate biomaterials and cell sources, and addressing the current challenges, researchers can create biomimetic vascular models that closely recapitulate the native vascular environment. The future of bioprinted thrombosis models lies in the integration of patient-specific data, real-time monitoring technologies, and advanced microfluidic platforms, paving the way for personalized medicine and targeted interventions. As the field of bioprinting continues to evolve, it is expected that these advanced vascular models will play an increasingly important role in unraveling the mysteries of thrombosis and improving patient outcomes.

## Funding

This work was supported by the National Health and Medical Research Council (NHMRC) of Australia (APP2003904 – L.A.J.); NSW Cardiovascular Capacity Building Program (Early-Mid Career Researcher Grant – L.A.J.; H22/98586 – K.L.); MRFF Cardiovascular Health Mission Grants (MRF2016165 – L.A.J.; MRF2023977 – L.A.J.) and MRFF Early to Mid-Career Researchers Grant (MRF2028865 – L.A.J.); NSW Government Boosting Business Innovation Program (BBIP) International Stream (L.A.J.); National Heart Foundation Vanguard Grant (106979 – L.A.J.); Office of Global and Research Engagement (International Sustainable Development Goal Program – L.A.J.). Lining Arnold Ju is a Snow Medical Research Foundation Fellow (2022SF176) and a National Heart Foundation Future Leader Fellow Level 2 (105863); Khoon Lim is an Australian Research Council Future Fellow (FT230100249).

## Ethics approval and consents to participant

Not applicable.

## CRediT authorship contribution statement

**Yanyan Liu:** Writing – review & editing, Writing – original draft, Investigation. **Tao Huang:** Writing – review & editing, Writing – original draft, Investigation. **Nicole Alexis Yap:** Writing – review & editing, Writing – original draft, Investigation. **Khoon Lim:** Writing – review & editing, Funding acquisition. **Lining Arnold Ju:** Writing – review & editing, Supervision, Funding acquisition, Conceptualization.

## Declaration of competing interest

All authors state they have no conflicts to declare.
